# ‘To be able to support her, I must feel calm and safe’: pregnant women’s partners perceptions of professional support during pregnancy

**DOI:** 10.1186/s12884-017-1411-8

**Published:** 2017-07-17

**Authors:** Caroline Bäckström, Stina Thorstensson, Lena B. Mårtensson, Rebecca Grimming, Yrsa Nyblin, Marie Golsäter

**Affiliations:** 1 University of Skövde, School of Health and Education, P.O. Box 408, SE-541 28 Skövde, Sweden; 2 Skaraborg Hospital Skövde, Woman, Child (K3), SE-541 85 Skövde, Sweden; 3 Jönköping University, School of Health and Welfare, CHILD-research group, Box 1026, SE-551 11 Jönköping, Sweden; 4 Närhälsan Skaraborg, Young Persons Clinic, SE-541 85 Skövde, Sweden; 5 Danderyd Hospital AB, Women’s care, Gynecology ward, SE-18288 Stockholm, Sweden

**Keywords:** Professional support, Partner, Expectant parent, Father, Pregnancy, Comother, Same-sex relationship, Childbirth, Phenomenography, Mother

## Abstract

**Background:**

Professional support does not always meet the needs of expectant fathers or co-mothers. The way in which professional support is offered during pregnancy varies internationally, depending on the country. In order to attain a greater understanding of partners’ experiences of professional support, it is necessary to further illuminate their perceptions of it. The aim of this study was therefore to explore pregnant women’s partners’ perceptions of professional support during pregnancy.

**Methods:**

Qualitative research design. Partners of pregnant women were interviewed during gestational week 36–38. Individual semi-structured interviews were used to explore the partners’ perceptions. The data was analysed using a phenomenographic approach. The study was performed in a county in south-western Sweden; the data collection was conducted from November 2014 to February 2015. Fourteen partners (expectant fathers and co-mothers) of women who were expectant first-time mothers with singleton pregnancies, were interviewed.

**Results:**

The findings of the study are presented through four descriptive categories: Ability to absorb adequate information; Possibility to meet and share with other expectant parents; Confirmation of the partner’s importance; and Influence on the couple relationship. Using a theoretical assumption of the relationship between the categories showed that the fourth category was influenced by the other three categories.

**Conclusions:**

The partners perceived that professional support during pregnancy could influence the couple relationship. The partners’ ability to communicate and to experience togetherness with the women increased when the expectant couple received professional support together. The support created also possibilities to meet and share experiences with other expectant parents. In contrast, a lack of support was found to contribute to partners’ feelings of unimportance. It was essential that the midwives included the partners by confirming that they were individuals who had different needs for various types of professional support. The partners perceived it easier to absorb information when it was adequate and given with a pedagogic that made the partners become interested and emotionally engaged.

## Background

Becoming a parent is one of the most emotional and life-changing experiences in a person’s life. Some people are well prepared to become parents, while others are not [1, 2]. Prepared or not, pregnancy, childbirth and the transition to becoming a parent should be experienced alongside the opportunity to receive professional support. Earlier research shows, however, that professional support does not always meet the needs of expectant fathers or co-mothers (henceforth ‘partners’) [1, 3, 4].

The way in which professional support is offered during pregnancy varies internationally, depending on the country. For example, some countries offers antenatal-education classes, while others do not [5]. However, international studies have found that professional support during pregnancy, leads to decreased numbers of pre-term births [6] as well as increased knowledge and better preparation for labour and birth [5] and for infant care [7]. In Sweden, partners are offered professional support together with the pregnant mothers-to-be; this support is for example offered during antenatal care, which is mostly provided by midwives [8]. In Sweden, antenatal units offer expectant parents six to nine antenatal visits with a midwife [9]; these visits could be described as health check-ups for detecting any pregnancy-related complications [10]. In addition to these visits, expectant parents (mainly first-time parents), in varying degrees are offered antenatal-education classes as well as some group-based lectures at hospitals.

Despite demonstrated benefits of professional support, some partners have expressed unmet support needs during pregnancy [4, 11, 12]. Partners tend to feel excluded from access to care [1, 12], even though they wish to be included and to have their support needs met [1, 11]. While preparing for childbirth, for example, partners worry about the expectant mothers and their babies [13]; they feel responsibility and want to be present during both pregnancy [14, 15] and childbirth [16]. Some partners find that pregnancy and the subsequent expectations of attending childbirth generate high levels of anxiety and fear [15–17] because they do not always know what is expected of them [18]. In addition, partners have been shown to be less involved in childcare if they were not educated, prepared or included in their child’s health, from pregnancy onwards [19].

Although earlier research has mostly focussed on expectant fathers, it is commonly known that when co-mothers are recognised in midwifery care, they feel valued for the qualities that separate them from others [4]. On the other hand, it is also known that co-mothers tend to feel excluded from professional support, when it focuses at mothers and fathers [20, 21]. This indicates that it is important to offer professional support for meeting the unique needs of the individual expectant partner, regardless of his/her gender [22]. While it is clear that professional support should include partners regardless of whether they are expectant fathers or co-mothers, the way in which professional support is currently offered to partners varies both nationally (in Sweden in this case) and internationally. This variation may indicate a knowledge gap concerning how professional support should be offered to meet support needs of expectant parents. Therefore it is important to attain a greater understanding of partners’ perceptions of professional support. The aim of this study was to explore pregnant women’s partners’ perceptions of professional support during pregnancy. Professional support is a broad concept that can be defined in various ways. In this study, professional support during pregnancy includes all different kinds of professional support partners are offered during pregnancy. Such support could come from: midwives at antenatal units or hospitals; obstetricians *or* psychologists for example. Moreover, the concept partner includes both expectant fathers and co-mothers in the present study.

## Methods

### Design and method description

The current study used the phenomenographic method which was originally developed by Marton [23]. Phenomenography is derived within the pedagogical rather than phenomenological traditions, knowledge/learning is the focus of studies that utilise this approach [24]. The central assumption of the phenomenographic approach is that people differ in their perceptions of phenomena; the intention of such studies is hence to discover the underlying structure of variance in the *perceptions* of a phenomenon, rather than in the phenomenon’s actual core [24]. These perceptions might be described by ‘how things are perceived or understood’ (i.e. the second-order perspective); this differs from the first-order perspective (‘how things really are’), which is the focus of other qualitative research methods [24]. In this study professional support is described as the phenomena.

### Settings and participants

This study was performed in a county in south-western Sweden with 280,000 inhabitants that consists of urban, suburban and rural districts. Approximately 2600 births occur annually at the county hospital’s labour ward. Within the geographical area of the study, expectant parents are offered professional support during prenatal assessments that take place at antenatal units (as described earlier). To a varying extent, parents are also offered antenatal-education classes in groups of six to eight couples four to five times during pregnancy. During these classes, midwives provide expectant parents with information about pregnancy, labour, breastfeeding, parenthood and relationships between spouses. Besides this, the midwives at the antenatal units refer expectant parents for additional assessments when necessary; these may include assessments by midwives/obstetricians at the hospital’s labour-ward unit, midwives who are trained and educated in handling fear of labour, and/or psychologists.

In the geographical area of the study, expectant parents are also offered professional support from the hospital via a lecture (Inspirational lecture). During the two-hour-long lecture, midwives provide expectant parents with information about normal labour and birth, with the intention of preparing parents for childbirth. The lecture provides information using elements of humorous role-playing with a focus both on the pregnant women and on the partners. The authors of this study have not participated in the professional support offered to the partners included in the study.

The inclusion criteria for this study included 1) partners of women who were expecting their first child with singleton pregnancies; further, they had to 2) intend to give birth at the county hospital and 3) both understand and speak Swedish. Midwives at various antenatal units asked partners to participate during a prenatal assessment in gestational week 25. Among the partners who accepted participation, strategic sampling was used to ensure variation in terms of age, place of residence (urban, suburban or rural districts), high school and/or university education, and the education moment(s) they were receiving (only antenatal education class, only the hospital lecture, or both). All participants were contacted by the first author via telephone. Prior to the commencement of the interviews, the informants had been provided with written information about the interviewer’s profession (midwife/PhD student).

### Data collection

Semi-structured interviews were conducted according to the phenomenographic tradition [23] in order to gather the partners’ perceptions of professional support during pregnancy. The interviews (which lasted 30–60 min) were held by the first author during gestational weeks 36–38. The interviews were conducted from November 2014 to February 2015 and were performed via telephone using an interview guide consisting of open-ended questions; the interviews were then audio-recorded and transcribed verbatim. The open-ended questions were the following: ‘What type of professional support have you received for childbirth and parenting?’ ‘How have you perceived the support?’ ‘What has the support meant to you?’ Probing questions such as ‘Could you explain further?’ were also used. Two pilot interviews were conducted prior to the first interview in order to test the procedure and to achieve coherence in performing the interviews. The results of the pilot interviews showed that the interview guide and technical equipment were suitable for responding to the aim of the study.

### Data analysis

Data analysis (shown in Table 1) was conducted according to the phenomenographic tradition described by Sjöström and Dahlgren [25]. The first author carried out the data analysis with continuous discussions with co-authors. During the analysis, all authors were reflective according to their preconceptions from earlier experiences of working with professional support.

**Table 1:** Phenomenographic data analysis according to Sjöström and Dahlgren [25], as used in the present study

1. Familiarisation	The 14 interviews, totalling 196 pages (A4), were read several times to become familiar with the data and to obtain a sense of the whole.
2. Compilation	The narratives from all respondents about professional support were gathered into statements; any significant statements that corresponded to the aim of the study were identified.
3. Condensation	Different statements were condensed in order to obtain a representative description of the partners’ perceptions.
4. Grouping	Statements that were similar to one another were grouped together. In total, 21 perceptions were found that were distinct from one another.
5. Comparison	Comparisons were made between the groups to find similarities and differences in their perceptions; in this way, distinct borders were found between groups.
6. Naming	Perceptions and the descriptive categories that emerged were discussed and named to highlight their essentials using a suitable level of abstraction.
7. Contrastive comparison	The descriptive categories that emerged were compared in terms of similarities and differences. Four descriptive categories and nine perceptions were found in the end, as shown in Fig. 1. The logical relationships between categories formed a hierarchical arrangement that was then presented as an ‘outcome space’.

### Ethical considerations

Prior to the data collection, all partners were provided with information (in both verbal and written format) on the study and about their right to withdraw their consent to participate at any time. Written informed consent was obtained from the partners, who then chose the interview times themselves. The confidentiality of any information they provided in the interviews was guaranteed; the findings are partly expressed using anonymous quotations to protect the speakers’ identities. The clinical head of service for the antenatal units granted access to undertake this study, which was approved by the Regional Ethical Review Board in Gothenburg, Sweden (Dnr: 197–14).

## Results

For the present study, a total of 38 partners were asked to participate, 20 of whom accepted. Using a strategic sampling, a total of 14 partners (both expectant fathers and co-mothers) were included in the study (Table 2). The specific number of expectant fathers and co-mothers will not be presented, because of an ethical consideration not to risk omitting the identity of any participant.

**Table 2:** Characteristics and professional support received during pregnancy (Antenatal education class *and/or* Inspirational lecture), among the participants

Age (yrs.), Range, (Mean)	26–39, (33.4)
Place of residence, n	
Urban district	7
Suburban district	3
Rural district	4
Education (yrs.), Range, (Mean)	12–18.5, (13.9)
Received professional support during pregnancy, n	
Antenatal Education Class (AEC)	11
Inspirational Lecture (IL)	12
None of AEC or IL	1

**Fig. 1:**
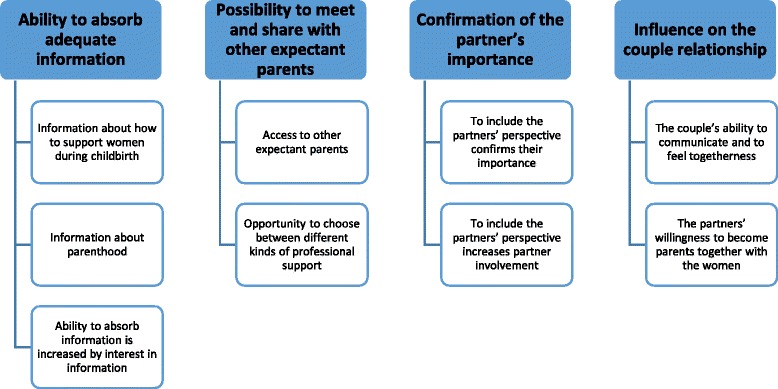
Overview of the four descriptive categories and their associated perceptions

The partners’ perceptions of professional support during pregnancy are presented in four descriptive categories: *Ability to absorb adequate information*; *Possibility to meet and share with other expectant parents*; C*onfirmation of the partner’s importance*; and *Influence on the couple relationship*. Each descriptive category had between two and three associated perceptions, as presented in Fig. 1; each perception was exemplified by using a partner’s direct quotes. The quotes have been edited slightly for clarity in English.

### Ability to absorb adequate information

This category stated that professional support could consist of information about how partners could support the pregnant women and take care of babies. The partners’ ability to absorb information increased when the information was pedagogically mediated with illustrative pictures. The partners’ interests in the information was affected by emotions, and they wanted the support to include the partners’ individual needs.

#### Information about how to support women during childbirth

Practical information and concrete advice about how to support women during childbirth was perceived as one aspect of the professional support. For example, the information could be about massage and breathing techniques or other ways to reduce and manage labour pain. The partners did express a few unmet information needs, however, such as the need for more information about childbirth complications (caesarean section, vacuum extraction, breastfeeding complications etc.) as well as information about how best to support women during the second stage of labour.

*… she might not want it like that right then, but in two minutes she might want it … and then I think that it’s best for me to just … not push it, but to do the right thing for her, even though she thinks it’s not so good …* (P9)

#### Information about parenthood

According to the partners, information about parenthood and how to take care of babies was essential in the professional support; they received this informative support particularly during antenatal-education classes. The information was mostly about breastfeeding without complications and how the parental couple should divide household tasks between them. When the information was about the latter, they perceived this as gender stereotyping as well as consisting of outdated admonitions about how parental couples should behave, which they perceived negatively. They wished to have more information about economic issues, parental leave, insurance and baby-related items that they needed to purchase. They also desired to have more knowledge about best how to understand a baby’s body language and behaviour and how to meet the baby’s needs.

[I wanted to know] *a little about what happens afterwards … how to act at home and … how often* [the baby] *should be bathed, and … things like that that we haven’t discussed.… I don’t know, I feel very uncertain about it … it’s more like you have to ask your parents … because I don’t feel prepared … about* [what happens] *afterwards, when the baby has been born and you’re at home … I feel like: ‘How will I do?’* (P14)

Because the partners also realised that parenthood might have an impact on the parental couple’s relationship, they wanted the information to be more about ways to keep their love alive after the child’s birth.

*... the everyday love life perhaps…* [how to] *make it bloom even though you have a little baby. That you do not spend all your love just at the baby and risk losing contact… That you do things together, even when the baby is with you… that you do not just stop living when you have a child. I think that’s very important.* (P 5)

#### Ability to absorb information is increased by interest in the information

When midwives illustrated the information using role-plays or PowerPoint slides, the partners perceived that their ability to absorb information increased; this enabled them to visually follow the information, which then contributed further to their understanding and learning. From a pedagogical aspect, this occurred because information that was communicated both verbally and via practical illustrations engaged several senses. Still, the partners did sometimes perceive that PowerPoint slides used as illustrations were old-fashioned pedagogy; the partners expressed the need for other practical illustrations as a way to engage them in their preparations for childbirth and parenting, such as illustrations in breathing techniques to use during labour and how to change diapers and bathe babies.

*How to change diapers and so on: I’ve never done that. I don’t know how to … wash or bathe* [babies. I think that they should offer] … *small courses … where you can learn* [how to do these things] *on performing dolls or pretend babies … that would have been interesting.…* (P14)

The partners perceived that being emotionally engaged and interested in the information that was provided contributed to their ability to absorb the information. Emotional engagement was created when the midwives used humour as a pedagogical approach, for example by telling parents how they could prepare for childbirth and parenting. They saw the humour that the midwives used in their lectures at the hospital (the Inspirational lecture) as being positive for learning. These midwives role-played and told stories about what could happen during labour. This humorous role-playing illustrated how a partner might feel or react, as well as how he or she could help the woman during labour. When the midwives used humour, it made the partners laugh, relax and feel more engaged in their childbirth preparations.

*They explain how it is.… The role-playing makes you feel* [emotions]*, and then* [the information] *was more fully absorbed;* [the childbirth] *felt safer afterwards.… Stating only facts doesn’t do much; but when your emotional life is involved, you absorb things better. You can laugh … you remember more that way.* (P9)

### Possibility to meet and share with other expectant parents

The partners perceived the ability to meet and share experiences with other expectant parents as positive. Lack of availability and opportunity to choose between different kinds of professional support, were perceived as negative.

#### Access to other expectant parents

The partners stated that professional support created opportunities to meet other expectant parents. These meetings provided the partners with opportunities to discuss various issues and experiences with others who were in the same situations. This support in particular was given during the antenatal-education classes. During these classes, the partners felt that it was important that the midwives create a permissive environment that would enable the partners to exchange their experiences with others. This was especially important when the classes included more than four to five parental couples, because large groups tended to prevent parents from talking. The partners perceived that when midwives altered between information and discussion sessions, it increased the partners’ willingness to engage in the discussions.

*It’s valuable because … it’s nice to have new acquaintances … who are our own age and in the same family situation.* [It is also] *valuable to have the opportunity to share our experiences in concrete terms – how it is now, and how you plan ahead – and you can talk about buggies and cars and … changing tables. This is valuable, because otherwise you …* [do not receive that information]*.* (P8)

#### Opportunity to choose between different kinds of professional support

The partners perceived it negative to be excluded from access to antenatal-education classes (or being limited in that access). Some partners had not been offered classes at all, while others had been offered classes but only during the day, which made their participation impossible because of work obligations. They desired to have access to different kinds of antenatal-education classes, with a focus on homogeneity between expectant parents. These suggestions included groups for expectant parents in same-sex relationships (this need was expressed by co-mothers), groups for partners only (i.e. excluding the pregnant women) or groups held by new parents. They wished to be able to choose educational formats based on their own interests and information needs. They perceived the lack of opportunity to participate as being unsatisfactory because of the missed opportunity to receive information and to meet other parents. Prenatal assessments with midwives at antenatal units could not replace antenatal-education classes.

*You’ve heard from others who found* [antenatal-education classes to be] *very good, funny, inspiring … it feels a bit sad that we weren’t offered* [classes] *… it feels sad that they* [professionals at antenatal unit] *took so easy at it* [excluding antenatal-education classes]. *You don’t get any help or anything from the antenatal unit … except for the controls, because then they do it … you have to manage very much on your own … you feel very excluded sometimes.* (P14)

### Confirmation of the partner’s importance

Partner’s importance could be confirmed when they received professional support; this support could also influence them to become more involved in asking questions about preparations for childbirth and parenting.

#### To include the partners’ perspective confirms their importance

When the professional support included the partners’ situations and individual needs, they perceived their importance to have been confirmed. This confirmation was also realised when a word such as ‘partner’ was used instead of ‘father’, because this confirmed and involved the co-mother partners. This support might consist of individual assessments with midwives or other healthcare professionals in the chain of antenatal and labour-ward care (e.g. psychologists or obstetricians). The partners perceived that when they had access to this support, they found that it was less intrusive of the women’s access to care. They expressed that an even greater access to professional support would create more opportunities for partners’ support needs to be met.

*The mood is very calm when you get there; it’s dimly lit and they have candles, and you sit and talk a lot; you can talk about virtually anything … you don’t need to be afraid to say anything; you don’t need to be afraid to say what you feel; you have to listen to* [the baby’s] *heart sounds … the debate feels more open: if you say something, you might as well speak what’s on your mind, because they’ll listen.…* (P 1)

As an example, the partners wanted the antenatal-education classes to include the role of the partner to a greater extent. In contrast, when the information was too focussed on the women’s situation and needs, this prevented the partners’ support needs from being met.

*If I am to be supportive and serve as a source of security for my partner, I must feel calm and safe, too.* (P8)

#### To include the partners’ perspective increases partner involvement

The partners perceived that their ability to be involved in the childbirth and parenting preparations (and their interest in the same) increased when that support included their perspective. This occurred when the midwives provided information about how partners could help and support women during labour. The information might be about massage and breathing techniques, pain-relief methods, or nutrition, or the best ways to help the women manage labour pain using mental strategies. The support was mostly received during the lecture at the hospital. Because the partners perceived that it was impossible to predict the experiences and outcomes of childbirth, they felt that it was difficult to prepare for childbirth. Still, they did thought that professional support could increase their feeling of being prepared for childbirth; this was because the increased knowledge created a sense of calmness and a feeling of security. Their self-esteem was strengthened when they were able to trust their own abilities.

[The increased knowledge] *makes me even more motivated to be there and to help and support … it is, of course, positive … it’s great to understand your role … in the process, too, because otherwise I think that the partner is often forgotten in this context, for obvious reasons … it turned out to be pretty clear that you could fulfil a very important function …* (P7)

### Influence on the couple relationship

Professional support increased the expectant parental couples’ abilities to communicate and to experience togetherness; it also contributed to the partners’ willingness to become parents and to take care of the baby together with the expectant mothers.

#### The couple’s ability to communicate and to feel togetherness

When the parental couple received professional support together, the support contributed to an increased ability to communicate within the couple; the couples perceived that this strengthened their feelings of togetherness. When the parental couple had different needs for preparing for childbirth and parenting, it was usually the pregnant women who had greater preparatory needs; these different needs could cause frustration within the couple. Professional support could decrease that frustration, however, because it made the partners understand their importance for each other. This situation made the partners more willing to communicate about how best to prepare for childbirth and parenting with the pregnant women.

*… It felt real before, too, but now it’s really … like a life-long* [commitment] *when you are about to become a parent… it has affected us in a good way … it sounds like a cliché, but it may be that we have matured in our relation with each other.* (P9)

#### The partners’ willingness to become parents together with the women

Professional support was perceived as contributing to the partners’ understanding of how best to prepare for parenthood; it made them more willing to prepare for parenting together with the pregnant women. The support influenced their willingness to become parents and to take care of the baby together with the mothers.

*You become more connected* [with your partner when both partners are] *going towards a* [common] *goal* [i.e. parenthood] *together.* (P1)

### The outcome space

**Fig. 2:**
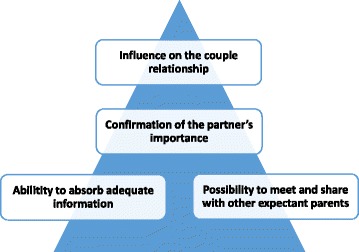
The findings in relation to the outcome space, and the hierarchical arrangement of the categories

When using phenomenography, the logical relationships between categories can be presented in an outcome space, where each category is related to the other categories; the categories together form part of a larger whole. Through this strategy, people’s various ways of thinking about their experiences of a phenomenon are presented [24]. For this study, a hierarchical arrangement arose during the data-analysis process. Through this arrangement, the partners’ understanding of their experiences of professional support during pregnancy was taken into account. Their understanding was related to the way in which the categories were related to one another and formed a larger, cohesive whole. The hierarchical arrangement shown in Fig. 2 is based on a theoretical assumption of the relationship between the categories.

Within this hierarchical arrangement, the category *Influence on the couple relationship* formed the top of the pyramid, because it was influenced by the other three categories. The category *Confirmation of the partner’s importance* formed the second level in the arrangement because of its connection to both the bottom and the top categories. The base of the arrangement was formed by the two underlying categories: *Ability to absorb adequate information* and *Possibility to meet and share with other expectant parents.* One example of this hierarchical arrangement might be that when professional support included adequate information with a high level of partner focus *(Ability to absorb adequate information)*, it confirmed the partners’ importance and created opportunities for them to become involved in the childbirth preparations (*Confirmation of the partner’s importance*)*.* The parental couple could then use their common experience of the support in their communications with each other. When the parental couple communicated about how to prepare for childbirth and parenting that communication led to a feeling of togetherness and the sense that their relationship had become strengthened *(Influence on the couple relationship).*

## Discussion

The most important finding of the current study show that the partners perceived that professional support during pregnancy positively affected the couple relationship. This effect occurred when the support confirmed the partner’s importance, consisted of adequate information or created access to other expectant parents. The partners perceived that their ability to communicate with the expectant mothers was increased by professional support and that it also increased the couples’ feeling of togetherness and the partners’ willingness to become parents, which is in agreement with earlier research on pregnant women [26]. The improved ability to communicate within the parental couple is a valuable benefit of the professional support, because it may help the couple to express their needs to each other. This highlights the valuable role that healthcare professionals play in their contact with expectant parents, since they can contribute to strengthened social support [27], for expectant parents [28, 29].

According to our findings, one way to support partners in their preparations for childbirth and parenting could be to inform them about ways to engage themselves. Earlier research has shown that such engagement might consist of searching for information and support in the preparations in order to adapt to the upcoming life changes that transition to parenthood includes [30]. Two ways for professionals to achieve this could be to pursue education about preparing for this transition [31] and create possibilities for social contacts with other expectant parents [26]. The partners included in the present study were offered education both through antenatal-education classes and through a lecture at the local hospital. Our findings showed that the partners perceived that their engagement in these education moments and their ability to absorb information were both increased by emotions, discussions and the pedagogically mediation made by the midwives (practical illustrative moments, for example). The education moments did also, according to the findings of the present study, create access to other expectant parents. Midwives who in general meet expectant parents can use this knowledge during educational sessions, to make it possible for partners to understand information and reflect on their forthcoming changing situations, both individually and with other expectant parents. Since, becoming a parent comprises a new role that will affect identity and the roles in the relationship of the parental couple [32]. The transition made by couples from partners to parents usually requires coping strategies [33, 34] and previous studies [35–38] have stressed the value in supporting expectant parents in their changing roles.

However, the co-mothers in the present study perceived that they sometimes were excluded by the professionals. This could happen when the midwives used words like ‘Fathers’ for example, which is earlier described as a source of making co-mothers feeling offended [20, 21], it highlights to co-mothers that they are different [21]. In contrast, the co-mothers in our study perceived that they were included and recognized when the midwives used words like ‘Partners’ instead. Additionally, the focus healthcare professionals have when giving professional support to expectant parents may leave out or include minorities, such as partners of pregnant women. Even though Sweden has been considered as one of the most gender equal countries in the world, the partners in the present study expressed that the professionals sometimes preserved traditional gender roles when talking about parenthood, for example, which is in line with earlier research [39, 40]. Therefore, partners regardless of their gender, should be visual in research concerning pregnancy and childbirth.

Further, the partners in the present study perceived that lack of professional support could contribute to feelings of unimportance. It is known since before that when partners feel unimportant, excluded from access to professional support, and/or unprepared to support a woman during labour, these factors may lead to negative effects for the partners and for the mothers and babies. Examples of these negative effects include childbirth fear [41] and the inability to support the woman during labour [16]. This highlights the importance of our findings, which are related to including partners (at least those who want to be included) in professional support. Healthcare professionals may use the results of the present study to increase their understanding of how best to meet the support needs of expectant parents, regardless of the context in which the expectant parents need support. However, offering right amount of professional support might be seen as a challenging act of balance, which should derive from the individual needs of the expectant parents. In addition, it is valuable to bear in mind the risk of over-professionalization, since over-use of risk labels might lead to professionals missing the value of seeing pregnancy and childbirth as a normal life event [42, 43]. An example of this might be professionals viewing pregnancy, childbirth and transition to parenthood as negatively influencing the parents’ wellbeing and couple relationship. Renfrew et al. [43], suggest that instead of focusing on risk identification, the primary focus of both care and research should be on optimising biological, psychological, social and cultural processes. With this perspective, the care should focus on the needs of individuals, to avoid viewing pregnancy and childbirth through a ‘risk lens’. Commonly, expectant parents use their already established social contacts to gain knowledge about how childbirth and parenthood influence couple relationship [28]. Therefore, professional support could be about creating opportunities to strengthen expectant parents’ social support, through parental education classes for example. Then, the expectant parents included in these classes could exchange experiences of the influences on couple relationship that comes with childbirth and parenthood.

Nevertheless, further research is still necessary to attain an even greater understanding of the concept of professional support during pregnancy; it would be valuable to evaluate the effects of such support. One such study direction could be to evaluate whether professional support can strengthen the couple relationship from a longitudinal perspective, since our findings showed that the partners perceived that professional support could strengthen the couple relationship before childbirth. It would also be valuable to explore new parents’ retrospective experiences of the professional support they had received during pregnancy. As well as explore midwives’ descriptions of how they work to meet expectant parents’ needs for professional support.

The authors of this article had varying experiences in working with professional support for expectant parents. One of the authors had none such experience. Therefore, pre-conceptions were discussed between authors both before, during and after the analysis process. Also, close reading of the transcript were current, with an attempt to ensure transparency and to obtain new insights and understanding of the phenomenon studied [44]. The results both confirmed some pre-conceptions and shed a new light on partners’ perceptions of professional support during pregnancy. As in the case of all qualitative studies, one should be cautious with transferability [45]. However, the findings of the present study might be transferred to other situations identified as similar, as they generally contribute a deeper understanding of the topic studied. Because many partners share the experience of pregnancy with the women, regardless of their country, gender or professional support available. However, the professional support offered to expectant parents in Sweden is relatively extensive, compared to other countries. Which could be considered as a study limitation when it comes to transferability. Nevertheless, humans vary in their ways of perceiving the world around them, it is important to consider the fact that it may be possible to find further perceptions when transferring the findings of phenomenographic research to similar settings. An earlier study has reported that when using phenomenographic analysis, it is common that no new perceptions will be revealed after ten to twelve interviews [46]. In the present study (which included fourteen partners), this assumption was strengthened by the result of the data analysis.

When conducting phenomenographic research, individual semi-structured interviews are the preferred method [25]. For this study, telephone interviews were conducted; when interviewing via telephone, the interviewer does not have the opportunity to analyse body language or facial expressions. The interviewer and the participant may be less affected by each other’s presence, however, which may increase the level of comfort for interviewer and participant alike, which thus may result in a more relaxed interview [47]. In this study, the interviewer had earlier experience with conducting telephone interviews and patient consultations, both of which were valuable during the data-collection process. All of the informants were able to express their experiences with the interviews afterwards; they stated that being interviewed via telephone made it possible to participate in the study, since it saved time, for example.

## Conclusions

The partners of pregnant women included in this study, perceived that professional support during pregnancy could influence the couple relationship. The partners’ ability to communicate and to experience togetherness with the women increased when the expectant couple received professional support together. The support created also possibilities to meet and share experiences with other expectant parents, which was perceived positive. In contrast, a lack of support was found to contribute to partners’ feelings of unimportance. It was essential that the midwives included the partners by confirming that they were individuals who had different needs for various types of professional support. The partners perceived it easier to absorb information when it was adequate and given with a pedagogic that made the partners become interested and emotionally engaged.

## Abbreviations

AEC: Antenatal Education Class
IL: Inspirational Lecture
